# Liver fibrosis and cirrhosis in the multi-omics era: mechanisms and therapeutic perspectives from human and animal models

**DOI:** 10.3389/fcimb.2025.1686649

**Published:** 2026-01-08

**Authors:** Weiwei Lu, Jun Xu, Yiting Xu, Yifeng Zhou, Shuping Que, Zhengtao Liu

**Affiliations:** 1 Key Laboratory of Artificial Organs and Computational Medicine in Zhejiang Province, Shulan International Medical College, Zhejiang Shuren University, Hangzhou, China; 2 Shulan (Hangzhou) Hospital, Shulan International Medical College, Zhejiang Shuren University, Hangzhou, China; 3 Division of Hepatobiliary and Pancreatic Surgery, Department of Surgery, First Affiliated Hospital, School of Medicine, Zhejiang University, Hangzhou, China; 4 NHC Key Laboratory of Combined Multi-Organ Transplantation, Key Laboratory of the Diagnosis and Treatment of Organ Transplantation, First Affiliated Hospital, School of Medicine, Zhejiang University, Hangzhou, China; 5 Key Laboratory of Organ Transplantation, Zhejiang Province, First Affiliated Hospital, School of Medicine, Zhejiang University, Hangzhou, China; 6 Ya-er-zhuang Clinics, Hangzhou, China

**Keywords:** liver fibrosis, liver cirrhosis, multi-omics, viral hepatitis, animal models, therapeutic response, mechanistic analysis

## Abstract

Liver fibrosis and cirrhosis are common outcomes of chronic liver diseases such as viral hepatitis, alcoholic liver disease, and non-alcoholic fatty liver disease. Despite diverse causes, they share core pathological features including hepatic stellate cell activation, extracellular matrix deposition, immune dysregulation, and metabolic alterations. Recent advances in multi-omics technologies—encompassing transcriptomics, proteomics, and metabolomics—enhance our understanding of the molecular and cellular mechanisms driving liver fibrosis. This review integrates findings from human studies and animal models, highlighting key pathological pathways and their interactions. Multi-omics analyses also clarify therapeutic mechanisms targeting oxidative stress, inflammation, and metabolic dysfunction. Cross-species comparisons confirm the translational relevance of animal models and underscore the value of multi-omics approaches in biomarker discovery and precision therapy development. Overall, these insights provide a systems-level understanding of liver fibrosis, facilitating advances in diagnosis and treatment strategies.

## 1 Introduction

Liver fibrosis is a pathological process of tissue remodeling characterized by the excessive accumulation of extracellular matrix (ECM) components, particularly type I and type III collagens, driven by persistent hepatic injury (Bataller and Brenner, 2005). In the early stages, fibrosis is potentially reversible through the removal or inhibition of the underlying insults, allowing for ECM degradation and partial restoration of hepatic architecture. However, sustained injury can lead to progressive fibrosis and ultimately cirrhosis, which is associated with severe complications such as hepatic failure, portal hypertension, and hepatocellular carcinoma (HCC) (Sun and Kisseleva, 2015). Globally, hepatitis B virus (HBV) and hepatitis C virus (HCV) remain the predominant etiological agents of liver fibrosis and cirrhosis, especially in Asia and Africa. Epidemiological studies estimate that approximately 3.8% of the global population (around 296 million individuals) are chronically infected with HBV, and an additional 58 million are living with chronic HCV infection, jointly accounting for nearly 57% of cirrhosis cases (Younossi et al., 2023). Meanwhile, the increasing prevalence of metabolic disorders and shifts in lifestyle lead to a rising burden of alcoholic liver disease (ALD) and non-alcoholic fatty liver disease (NAFLD), including its progressive form, non-alcoholic steatohepatitis (NASH) (Riazi et al., 2022; Åberg et al., 2024).

Liver fibrosis is a highly dynamic and spatially heterogeneous process orchestrated by complex multicellular interactions. Key cellular players include hepatic stellate cells (HSCs), Kupffer cells (KCs), liver sinusoidal endothelial cells (LSECs), and various infiltrating immune cells (Acharya et al., 2021). Under conditions of chronic inflammation or metabolic stress, quiescent HSCs become activated and transdifferentiate into myofibroblast-like cells, serving as the principal source of ECM production. Kupffer cells release proinflammatory cytokines such as TNF-α and IL-1β, promoting immune cell recruitment and synergizing with HSCs to exacerbate fibrogenesis. Concurrently, LSEC dysfunction and capillarization further enhance HSC activation and alter the hepatic microenvironment (Gao et al., 2024). Collectively, liver fibrosis results from temporally and spatially coordinated actions among HSCs, KCs, LSECs, and immune cells. Traditional single-omics approaches prove inadequate in fully deciphering this intricate pathophysiological network, thereby highlighting the critical need for integrative multi-omics strategies. Given the rapidly growing body of research on liver fibrosis, it is imperative to systematically categorize and elucidate existing findings to achieve a more comprehensive understanding of the disease (Zhu et al., 2024). Although the literature in this field is abundant, a thorough and structured synthesis remains lacking. This highlights the necessity for organized classification and integrative evaluation, which can effectively inform future research directions and clinical decision-making.

In recent years, high-throughput omics technologies—including transcriptomics, proteomics, and metabolomics—provide invaluable insights into the molecular mechanisms underlying liver fibrosis. In a chemically induced murine fibrosis model, Zhang et al. conducted integrated RNA sequencing and mass spectrometry-based proteomics, identifying 1,224 differentially expressed genes and 302 differentially expressed proteins. Notably, 69 molecules showed concordant changes at both transcriptomic and proteomic levels, implicating pathways involved in metabolism, detoxification, and ECM remodeling. These findings underscore the limitations of single-omics methods in capturing the complexity of fibrosis biology (Zhang et al., 2023). Furthermore, Yang et al. performed dual-omics analyses in human liver tissues and plasma samples from patients with HBV, ALD, and NAFLD. Across 330 clinical samples, they uncovered common fibrotic signatures involving ECM remodeling, immune activation, and metabolic reprogramming. Importantly, they identified 132 circulating proteins as candidate biomarkers for advanced fibrosis, with an area under the ROC curve (AUC) of 0.89, outperforming the widely used FIB-4 model (AUC 0.85) (Yang et al., 2025). Collectively, the integration of multi-omics datasets across transcriptomic, proteomic, and metabolic layers enabled the construction of a systems-level regulatory atlas of liver fibrosis. These integrative approaches advanced our understanding of disease heterogeneity and offered a foundation for developing precision diagnostic and therapeutic strategies.

This body of research encompasses both human and animal studies. In human cohorts, multi-omics approaches reveal distinct yet overlapping molecular features of fibrosis induced by HBV and HCV, including differential activation of signaling pathways, remodeling of the immune microenvironment, and metabolic reprogramming—particularly in bile acid and lipid metabolism. These findings underscore the heterogeneous nature of fibrosis across etiologies and patient populations. In parallel, animal models provide mechanistic insights and served as platforms for therapeutic evaluation. Carbon tetrachloride (CCl_4_)-induced liver fibrosis models were employed to investigate the effects of various pharmacological agents and natural compounds, such as gypenoside, Zi Qi, metformin, and kaempferol, which exhibited antifibrotic properties primarily through antioxidative, anti-inflammatory, and immunomodulatory mechanisms. The bile duct ligation (BDL) model was utilized to examine the regulatory impact of Ampelopsis grossedentata tea on bile acid dysregulation. In HBV-infected animal models, studies explored adeno-associated virus (AAV)-mediated gene delivery systems to dissect virus-specific fibrogenic mechanisms. Additional research focused on the role of ubiquitination in modulating fibrotic progression within the CCl_4_ model, further expanding our understanding of post-translational regulatory networks in fibrosis.

Although numerous reviews have explored specific molecular mechanisms of liver fibrosis or focused on the application of individual omics technologies, a comprehensive, systematic overview that spans multi-omics platforms and simultaneously integrates data from both human and animal models is still lacking. This review fills this gap for the first time. We focus on how multi-omics technologies—including cutting-edge approaches such as single-cell and spatial omics—work in synergy to systematically decipher the complex pathogenesis of liver fibrosis and cirrhosis. More importantly, through comparative analysis of multi-omics landscapes derived from human specimens and animal models, we clearly define the strengths and limitations of animal models at different stages of disease progression and, based on this, propose a novel analytical framework to guide future translational research. Therefore, the unique value of this review lies in its provision of a highly integrated and translationally oriented “multi-omics era” blueprint for liver fibrosis and cirrhosis research, bridging basic and clinical studies.

## 2 Mechanisms of liver fibrosis: insights from multi-omics approaches

Liver fibrosis is a common pathological outcome of various chronic liver diseases, including viral hepatitis, alcoholic liver disease (ALD), and non-alcoholic fatty liver disease (NAFLD) (Bataller and Brenner, 2005). Although these conditions ultimately lead to fibrotic remodeling, they differ significantly in the pathways driving disease progression, clinical trajectories, underlying molecular mechanisms, key effector molecules involved, and interactions with environmental and host factors. For instance, viral hepatitis—primarily caused by hepatitis B virus (HBV) and hepatitis C virus (HCV)—is largely associated with immune-mediated liver injury and chronic inflammation that promotes fibrogenesis (Guidotti and Chisari, 2006). In contrast, alcohol-induced liver damage is closely linked to oxidative stress and proinflammatory signaling cascades (Cederbaum et al., 2009). Meanwhile, NAFLD is tightly associated with metabolic syndrome and is characterized by a complex interplay between lipid accumulation, insulin resistance, and inflammation (Friedman et al., 2018; Henry et al., 2025). These etiological distinctions underscore the critical importance of developing targeted, etiology-specific therapeutic strategies, rather than relying on a one-size-fits-all approach. A comprehensive understanding of the molecular and cellular basis of fibrosis across different causes is therefore essential for advancing precision medicine in hepatology.

Given the multifactorial nature of liver fibrosis, both human and animal studies are indispensable for elucidating its pathogenesis. Human studies provide valuable insights into the clinical course, epidemiological features, and associated biomarkers of fibrosis. In this review, we first summarize the key findings from cohorts of HBV- and HCV-related liver disease patients based on **Table 1**, including patterns of fibrosis progression, histopathological features, molecular markers, and disease-specific pathways identified through high-throughput omics approaches.

**Table 1:** Multi-omics studies on human hepatic fibrosis and cirrhosis.

Author, country, publication year (ref)	Patient type	Gender (male)/Age	Hepatic biochemical profile^a^	Assays/platform	Omics	Case vs. control	Comparison	Statistics	Major findings	Key pathway	Validation	Database number
Song, China, 2024 (Song et al., 2024)	HC	NA	NA	Gene Expression Microarray/Affymetrix Human Genome U133 Plus 2.0 Array(GPL570)	Transcriptomics	122 vs.6	HBV vs.HC	Student’s t-test/Mann-Whitney test/ANOVA	1.CD8A may be involved in the progression of CHB via regulation of immune functions.	1.Cytokine-Cytokine Receptor Interaction	Immune Cell Infiltration Analysis	GSE83148
	HBV									2. Viral Protein Interaction with Cytokine and Cytokine Receptor		GSE84044
	HBV-LF									3. Chemokine Signaling Pathway		
						71(F1:20, F2:33, F3:18, F4:10) vs.43(F0)	HBV vs.HBV-LF		2.CCL20 and CD8A were significantly upregulated in HBV and HBV-LF samples, increasing gradually with the stage of histological fibrosis.	1.Viral Protein Interaction with Cytokine and Cytokine Receptor		
										2.Chemokine Signaling Pathway		
										3.Cytokine-Cytokine Receptor Interaction		
Dong, China, 2023 (Dong et al., 2023)	HC	1(20%)/50.0(42.0,53.0)	ALT:11.8 (11.00, 18.35)	RNeasy Mini Kit and RNA-Seq/Illumina NovaSeq 6000	Transcriptomics	6 vs.5	HBV late-stage vs.HC	DESeq2	1.SP1, RELA, and NFKB1 were identified as key regulators in regulating the liver cirrhosis process.	1.Focal Adhesion	RT-qPCR	GSE29868
			AST:34.46 (12.95, 18.6)							2.PI3K–Akt Signaling Pathway		
			TBIL:8.9 (7.5, 12.9)							3.Regulation of Actin Cytoskeleton		
			DBIL:1.5 (1.3, 2.3)									
			TBA:2.3 (1.5, 4.1)									
			CHE:8438 ± 1573.19									
			ALB:37.13 ± 5.73									
	HBV late-stage patients	5(83%)/44.5(43.25,46.5)	ALT:23.05 (20.10, 51.25)	LC-MS/MS/timsTOF and Evosep One	Proteomics	6 vs.5	HBV late-stage vs.HC	Student’s t-test/Mann–Whitney U test	2.The modules of the cytokine-signaling pathway, actin cytoskeleton organization, and signaling of Rho-GTPase may play significant roles in the development of liver fibrosis.	1.Phagosome	1. WB	PXD040012
			AST:149.97 (43.02, 140.68)							2.Cell Adhesion Molecules	2. IF	
			TBIL:84.65 (49.0, 109.95)							3.Focal Adhesion		
			DBIL:20.95 (12.35, 50.4)									
			TBA:82.5 (47.3, 349.8)									
			CHE:3715 ± 660.99									
			ALB:36.4 ± 3.16									
Shanmuganathan, Canada, 2021 (Shanmuganathan et al., 2021)	HCV late-stage(F2-F4)	9(82%)/63 ± 11	HCV early-stage: GGT:132 ± 125	LC-MS/MSI-CE-MS and NMR	Metabolomics	20(F0:5, F1:4, F2:5, F3:2, F4:4) vs.14	HCV vs.Non-HCV	Student’s t-test/ANOVA	1.Oxo-proline concentrations were higher in HCV patients with liver fibrosis as compared to non-HCV controls, reflecting elevated oxidative stress and glutathione depletion from chronic inflammation.	1.Glycine, serine and threonine metabolism	Cross-Platform Comparison	MetaboLights
			TBIL:14.8 ± 7.5							2.Phenylalanine, tyrosine and tryptophan biosynthesis		
			A2M:3.4 ± 1.2							3.Valine, leucine and isoleucine biosynthesis		
			Hb:1.09 ± 0.51							4.Phenylalanine metabolism		
			ApoA1:1.44 ± 0.11									
			ALT:82 ± 55									
	HCV early-stagee(F0-F1)	8(89%)/57 ± 10	GGT:76 ± 77			9(F0:5, F1:4) vs.11(F2:5, F3:2, F4:4)	HCV early-stage vs.HCV late-stage		2.The serum metabolites choline, histidine, proline, and 2-hydroxybutyric acid were elevated in HCV patients with more advanced liver fibrosis severity.			
			TBIL:11.8 ± 6.8									
			A2M:4.4 ± 1.2									
			Hb:1.40 ± 0.75									
			ApoA1:1.40 ± 0.30									
			ALT:52 ± 36									
	Non-HCV	10(77%)/44 ± 15	GGT:29 ± 26 U/L						3.The choline to uric acid ratio was found to optimally differentiate between late (F2-F4) and early (F0-F1) stages of liver fibrosis.			
			TBIL:11.0 ± 6.3									
			A2M:1.82 ± 0.56									
			Hb:1.03 ± 0.39									
			ApoA1:1.36 ± 0.20									
			ALT:24.1 ± 8.4									
Cano, Spain, 2017 (Cano et al., 2017)	F0	67.9%/64.8 ± 8.5	ALT:113 ± 120 U/l	UHPLC-MS/TargetLynx	Metabolomics	134 (F0:81, F1:53) vs.69 (F2:41, F3:22, F4:6)	Slow fibrosers vs.Rapid fibrosers	OPLS-DA/ANOVA/Student’s t-test	1.TCDCA, GCA, and SM(d18:0/18:0), which rose very significantly along with the severity of liver fibrosis, were the metabolites that better discriminated between rapid and slow fibrosers in both univariate and multivariate analyses.	1.Bile Acid Biosynthesis and Secretion	1.LDA	GSE199732
			GGT:234 ± 400							2.Sphingolipid Metabolism	2.LOOCV	
			ALP:391 ± 407							3.Glycerophospholipid Metabolism		
			TBIL:20.5 ± 18.8									
	F!	60.0%/65.6 ± 7.5	ALT:144 ± 194						2.The diagnostic accuracy of this non-invasive technique is excellent compared with the most widely used and validated tests and opens new possibilities to avoid invasive procedures.			
			GGT:313 ± 518									
			ALP:370 ± 271									
			TBIL:18.8 ± 12.0									
	F2	68.3%/64.2 ± 9.1	ALT:148 ± 111									
			GGT:365 ± 404									
			ALP:466 ± 611									
			TBIL:25.7 ± 18.8									
	F3	54.5%/68.4 ± 7.3	ALT:173 ± 132									
			GGT:651 ± 1013									
			ALP:683 ± 590									
			TBIL:34.2 ± 30.8									
	F4	33.3%/67.5 ± 8.9	ALT:183 ± 68									
			GGT:970 ± 863									
			ALP:784 ± 234									
			TBIL:54.7 ± 56.4									

a Hepatic biochemical profile is presented as either “(median, IQR)” or “(mean ± SD)”.

Units: ALT, U/L; AST, U/L; CHE, U/L; ALB, g/L; TBIL, μmol/L; DBIL, μmol/L; TBA, mol/L; GGT, U/L; A2M, g/L; Hb, g/L; ApoA1, g/L; ALP, mg/dl.

ALB, albumin; ALT, alanine aminotransferase; ALP, alkaline phosphatase; A2M, alpha-2-macroglobulin; ANOVA, analysis of variance; ApoA1, apolipoprotein a1; AST, aspartate aminotransferase; CHB, chronic hepatitis B; CHE, cholinesterase; DBIL, direct bilirubin; GGT, gamma glutamyl transpeptidase; Hb, Hapatoglobin; HBV, hepatitis B viru; HC, healthy control; HCV, hepatitis C viru; IF, Immunofluorescence; LDA, linear discriminant analysis; LF, liver fibrosis; LOOCV, leave-one-out cross-validation; NA, not available; NC, negative control; NMR, maximum neighborhood component; qRT-PCR, quantitative real-time PCR; TBA, total bile acid; TBIL, total bilirubin; TOF, time-of-flight; UHPLC, Ultra-High Performance Liquid Chromatography; WB, western blot; wks, weeks.

### 2.1 Transcriptomic discoveries in hepatic fibrosis

Transcriptomic profiling is a pivotal strategy for elucidating gene expression reprogramming during HBV-associated liver fibrosis, and is extensively employed across both clinical and experimental studies. High-throughput RNA sequencing of liver tissues or peripheral blood samples enables the comprehensive identification of differentially expressed genes (DEGs) across disease stages and histological conditions. Subsequent bioinformatic analyses facilitate the characterization of regulatory networks and signaling cascades implicated in fibrogenesis. Notably, canonical fibrosis-related pathways such as TGF-β, NF-κB, and PI3K-Akt were consistently enriched across multiple datasets, underscoring their central roles in the pathogenesis of HBV-induced fibrosis (Trehanpati et al., 2012; Kan et al., 2017; Xie et al., 2020). Moreover, transcriptomic data identified putative transcriptional regulators—including STAT3 and CEBPB—which may mediate key processes such as inflammation, hepatic stellate cell activation, and extracellular matrix accumulation in the setting of persistent viral infection (Ren et al., 2023; Tang et al., 2024).

Dong et al. performed RNA sequencing on primary HSCs isolated from patients with HBV-related cirrhosis, identifying 2,156 differentially expressed genes (DEGs), including notable upregulation of COL1A1, TIMP1, ACTA2, and CTGF—genes essential for ECM production and HSC activation (Dong et al., 2023). Functional enrichment revealed significant involvement of these genes in Cell Adhesion Molecules, ECM–receptor interaction, Regulation of Actin Cytoskeleton, PI3K-Akt signaling, and focal adhesion pathways, emphasizing the structural and signaling reorganization characteristic of activated HSCs.

In a complementary study, Song et al. utilized integrated bioinformatics analysis of GEO datasets (GSE83148 and GSE84044), identifying 34 robust DEGs associated with disease progression from chronic hepatitis B (CHB) to HBV-induced liver fibrosis (HBV-LF). Among these, CCL20 and CD8A were highlighted as potential diagnostic biomarkers, with expression levels increasing alongside histological fibrosis grades (Song et al., 2024). Functional annotation indicated their roles in Cytokine–Cytokine receptor interaction, Viral Protein Interaction with Cytokine and Cytokine Receptor and Chemokine Signaling Pathway, underscoring the pivotal contribution of immune modulation in the transcriptional landscape of HBV-LF.

### 2.2 Proteomic discoveries in hepatic fibrosis

With the continuous advancement of high-throughput mass spectrometry technologies, proteomics emerges as a powerful tool for investigating the dynamic changes in protein expression and regulatory mechanisms involved in HBV-associated liver fibrosis. By comprehensively identifying and quantifying proteins in liver tissues or serum samples, researchers are able to construct protein expression profiles across different stages of fibrosis and identify differentially expressed proteins (DEPs) closely associated with disease progression. These studies consistently show that multiple key signaling pathways are repeatedly enriched in the context of HBV-induced fibrogenesis. Moreover, proteomic analyses reveal the aberrant expression of several potential functional proteins—such as GSTP1, CAT, and PRDX1—which are implicated in hepatic stellate cell activation, extracellular matrix accumulation, and vascular dysfunction (Kan et al., 2017). These findings not only deepen our understanding of the molecular mechanisms underlying HBV-related fibrosis but also provide valuable targets for early diagnosis and therapeutic intervention.

Proteomic profiling by Dong et al. identified 711 differentially expressed proteins (DEPs), with three proteins closely related to liver fibrosis—TIMP1, COL1A1, and ACTA2—being highly expressed in the liver fibrosis group (P<0.05). These proteins are primarily enriched in pathways regulating ECM architecture, cytoskeletal organization, and cell–matrix interactions. KEGG analysis confirmed enrichment in Phagosome, Cell Adhesion Molecules, Focal Adhesion, Natural Killer Cell-Mediated Cytotoxicity and Leukocyte Transendothelial Migration, corroborating the transcriptomic findings and emphasizing the central role of ECM remodeling in fibrosis progression (Dong et al., 2023).

### 2.3 Metabolomic discoveries in hepatic fibrosis

As a powerful tool for profiling dynamic changes in small-molecule metabolites, metabolomics play an increasingly important role in elucidating the mechanisms of hepatitis C virus (HCV)-related liver fibrosis (Yang et al., 2023b). Chronic HCV infection induces not only persistent inflammation but also widespread disturbances in host energy, amino acid, and lipid metabolism. These metabolic alterations provide a unique opportunity for identifying noninvasive biomarkers and clarifying the molecular basis of fibrosis progression (Ferrasi et al., 2023).

In a metabolomics study by Shanmuganathan et al., polar serum metabolites in HCV-infected patients were profiled using a dual-platform approach combining MSI-CE-MS and NMR. The study identified several metabolites associated with liver fibrosis severity. Levels of histidine, choline, and proline significantly increased with fibrosis progression (Shanmuganathan et al., 2021). Elevated histidine suggests enhanced oxidative stress and immune activation; choline accumulation reflects disrupted membrane lipid metabolism and impaired VLDL synthesis—both typical features of HCV infection. Proline, a precursor of collagen, is directly linked to extracellular matrix (ECM) accumulation. Importantly, the choline/uric acid ratio demonstrated strong diagnostic performance (AUC = 0.848) and positively correlated with liver stiffness measured by Fibroscan (r = 0.606), indicating its potential as a noninvasive marker for fibrosis progression. Enrichment analysis revealed that these metabolites are involved in pathways such as glycine-serine-threonine metabolism, phenylalanine metabolism, aromatic amino acid biosynthesis, and branched-chain amino acid biosynthesis, highlighting the critical role of amino acid metabolic reprogramming in HCV-related liver fibrosis.

In a large-scale metabolomic study involving 203 HCV-infected liver transplant recipients one year post-surgery, Cano et al. identified 444 metabolites using UHPLC-MS (Cano et al., 2017). They developed a lipid-based biomarker panel consisting of two sphingomyelins (SM) and two phosphatidylcholines (PC) to distinguish rapidly progressive fibrosis (F2–F4) from slowly progressive fibrosis (F0–F1), achieving excellent diagnostic performance (AUROC = 0.92, sensitivity 71%, specificity 85%, accuracy 84%). Key metabolites—SM(d18:2/16:0), SM(38:1), PC(16:0/16:0), and PC(16:0/18:0)—were significantly elevated in the rapid progression group. Additionally, the study found that bile acids and aromatic amino acids increased with fibrosis severity, while the BCAA/ArAA ratio decreased, indicating disrupted amino acid metabolism. Pathway enrichment analysis revealed that dysregulated sphingolipid and glycerophospholipid metabolism, along with impaired bile acid synthesis and secretion, may contribute to hepatocyte injury and hepatic stellate cell (HSC) activation in rapidly progressive fibrosis.

HCV-associated liver fibrosis exhibits a characteristic pattern of metabolic reprogramming, with systemic disruptions observed across amino acid, lipid, and bile acid metabolic axes. These alterations reflect the liver’s adaptive imbalance under chronic HCV infection, particularly in terms of energy metabolism, oxidative stress response, and membrane lipid remodeling, while also uncovering critical metabolic links between hepatic stellate cell (HSC) activation, extracellular matrix (ECM) deposition, and immune-inflammatory regulation. The metabolic signatures identified through multi-platform metabolomic profiling and enrichment analysis demonstrate strong discriminatory performance and biological relevance, with some outperforming conventional clinical parameters in fibrosis staging. As transcriptomic and proteomic data become increasingly integrated, future studies may establish multi-omics-based frameworks that comprehensively assess etiology, disease progression, and treatment response. This provides more precise tools and mechanistic insights to support early diagnosis, longitudinal monitoring, and personalized intervention in HCV-related liver fibrosis.

### 2.4 Core mechanism model under omics integration

With the rapid advancement of high-throughput technologies, multi-omics integration emerges as a powerful approach to elucidate the complex mechanisms of liver fibrosis (Ægidius et al., 2020). By systematically analyzing transcriptomic, proteomic, and metabolomic data, researchers gain a comprehensive understanding of the dynamic transitions from normal liver to fibrosis and ultimately cirrhosis. These integrative studies not only reveal temporal and spatial molecular alterations but also identify potential biomarkers and therapeutic targets across disease stages (Li et al., 2025).

In HBV-related liver fibrosis, multi-omics analysis reveals several key regulatory factors and signaling pathways. Two key molecules identified in the study are PKM2 and EHD2. Both molecules are significantly upregulated at both the mRNA and protein levels and are confirmed through protein interaction network analysis (such as MCODE clustering) to be involved in the key biological processes of liver fibrosis. PKM2 is a key enzyme in the glycolysis pathway, and its upregulation in liver fibrosis may be closely related to cellular metabolic reprogramming, thereby affecting the activation of hepatic stellate cells and fibrous deposition. EHD2 is involved in membrane transport regulation and may promote the development of liver fibrosis by regulating cytoskeleton remodeling and cell adhesion-related processes (Dong et al., 2023). CCL20 is an important member of the CC chemokine family, which primarily functions through its receptor CCR6 and is involved in the chemotaxis and activation of inflammatory cells. In HBV-related liver fibrosis (HBV-LF), the high expression of CCL20 may be closely related to the infiltration of immune cells in the liver and inflammatory responses, thereby affecting the occurrence and development of liver fibrosis. CD8A is an important co-receptor on the surface of T cells and is involved in T cell-mediated immune responses. In the process of HBV infection and liver fibrosis, the high expression of CD8A may be related to cell-mediated immune defense and the regulation of T cell functions (Song et al., 2024).

Metabolomics studies in hepatitis C virus (HCV)-related fibrosis uncover a distinct metabolic reprogramming landscape that correlates with fibrosis progression. Using MSI-CE-MS and nuclear magnetic resonance (NMR) platforms, elevated levels of histidine, choline, and proline—metabolites associated with oxidative stress, membrane synthesis, and collagen production—were observed across fibrosis stages (Shanmuganathan et al., 2021). Pathway enrichment analysis pointed to glycine-serine-threonine metabolism and branched-chain amino acid biosynthesis as central metabolic nodes. Furthermore, in HCV-recurrent liver transplant recipients, lipidomic profiling distinguished rapid from slow fibrosers based on altered levels of sphingomyelins (e.g., SM[d18:2/16:0]) and phosphatidylcholines (e.g., PC[16:0/16:0]), implicating disrupted bile acid synthesis and lipid metabolism in aggressive ECM deposition (Cano et al., 2017).

**Figure 1:**
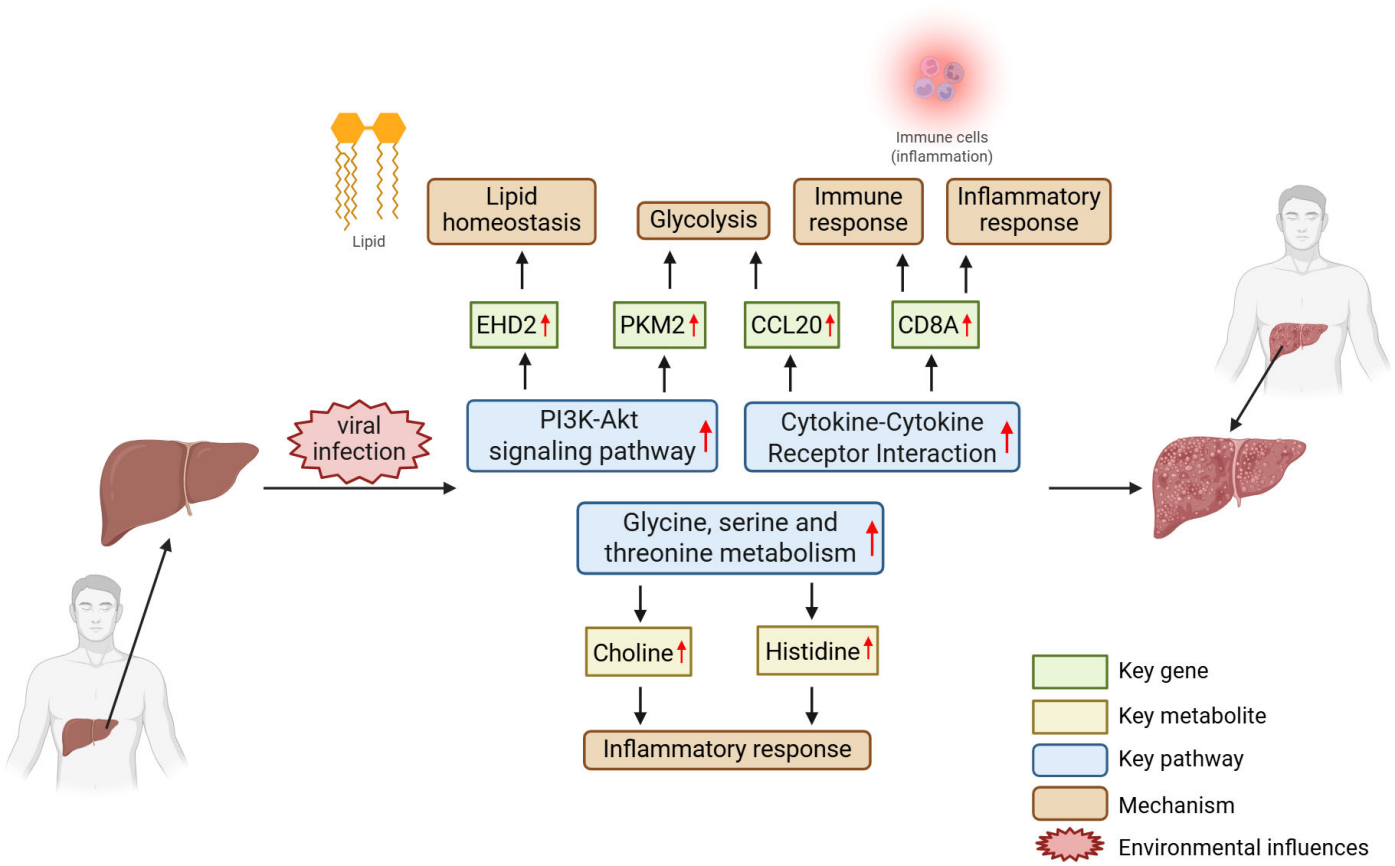
Multi-omics mechanisms in liver fibrosis and cirrhosis progression after viral infection. Green square boxes and corresponding red arrows indicate key genes significantly upregulated during liver fibrosis; yellow square boxes and corresponding red arrows represent key metabolites upregulated during liver fibrosis; blue square boxes denote critical signaling pathways activated during liver fibrosis; and brown square boxes highlight core mechanisms that play essential roles after fibrosis formation. Biorender was applied as a tool to generate this figure.

From a systems biology perspective, integrating multi-omics data allows the construction of a dynamic regulatory atlas of liver fibrosis. At the transcriptional level, SP1, NFKB1, and downstream cytokines (e.g., CCL20, CD8A) initiate hepatic stellate cell (HSC) activation and mediate immune crosstalk (Dong et al., 2023; Song et al., 2024). At the proteomic level, cytoskeletal proteins (e.g., TAGLN, ACTA2) and ECM structural components (e.g., LUM) drive matrix remodeling (Dong et al., 2023). At the metabolic level, imbalances in amino acid, sphingolipid, and bile acid metabolism provide both the energy and modulatory environment for fibrogenic progression (Cano et al., 2017; Shanmuganathan et al., 2021). This integrative view captures the continuum from quiescent to activated HSCs and reveals stage-specific intervention nodes across molecular layers. **Figure 1**, generated using BioRender, schematically illustrates the multi-omics interconnections among key genes, metabolites, and signaling pathways implicated in viral infection–induced liver fibrosis, offering a conceptual framework for elucidating its pathogenesis.

Multi-omics integration provides a high-resolution, system-level strategy for dissecting the mechanisms of liver fibrosis (Li et al., 2025). Current evidence highlights disease evolution from molecular events to systems disruption, with convergence on key signaling axes such as PI3K-Akt, TGF-β, and Rho-GTPase. Moving forward, these core networks should be leveraged to develop early diagnostic panels and personalized intervention strategies based on both human cohorts and preclinical models, paving the way for precision medicine in chronic liver disease.

## 3 Therapeutic responses in animal models: an integrated multi-omics perspective

In the study of antifibrotic therapies, animal models serve as essential platforms for evaluating therapeutic responses, elucidating disease mechanisms, and identifying molecular targets (Tsuchida and Friedman, 2017). Different models are designed to mimic distinct types of liver injury and fibrotic progression. For instance, carbon tetrachloride (CCl_4_) and thioacetamide (TAA)-induced models are commonly used to replicate fibrosis driven by toxic hepatic injury. HBV transgenic mice and AAV-HBV infection models are widely applied to study chronic inflammation-induced fibrogenesis in viral hepatitis. In contrast, the bile duct ligation (BDL) model simulates cholestatic liver fibrosis by obstructing bile flow, causing intrahepatic bile duct dilation, cholestasis, and fibrotic remodeling, making it a classic model for studying biliary-type fibrosis and therapeutic interventions.

With the rapid advancement of high-throughput omics technologies, integrated transcriptomic, proteomic, and metabolomic analyses open new avenues for systematically dissecting therapeutic responses at multiple biological levels. Transcriptomics enables the comprehensive profiling of differentially expressed genes (DEGs) before and after treatment, revealing dynamic changes in regulatory networks and signaling pathways. For example, studies demonstrate that antifibrotic interventions can restore the expression of key fibrogenic pathways such as TGF-β, NF-κB, and JAK-STAT, suggesting that these treatments may alleviate fibrosis by suppressing inflammation and hepatic stellate cell activation. Proteomics further validates the modulation of key proteins—such as GSTP1, CAT, and PRDX1—involved in signaling transduction, oxidative stress response, and extracellular matrix metabolism (Kan et al., 2017). In addition, metabolomics studies in HBV or CCl_4_-induced models reveal that therapeutic interventions can regulate core metabolic pathways, including bile acid metabolism, lipid metabolism, and amino acid cycling, providing direct evidence for metabolic reprogramming during fibrosis reversal (Lan et al., 2022).

Through the integration of multi-omics approaches, researchers can more comprehensively evaluate therapeutic efficacy, identify potential biomarkers, screen for sensitive prognostic indicators, and even predict individual responses to treatment (Baião et al., 2025). This systems-level strategy provides a strong theoretical and data-driven foundation for the development of precise and effective antifibrotic therapies in the future (Li et al., 2025). **Supplementary Table 1** summarizes the major findings from multi-omics studies in animal models of liver fibrosis and cirrhosis, including key genes, metabolites, and signaling pathways, as well as their roles in elucidating disease mechanisms and identifying potential therapeutic targets.

### 3.1 Transcriptomics discoveries in animal models

Transcriptomics is a core approach in hepatic fibrosis research, offering critical insights into gene expression reprogramming and regulatory networks. Through transcriptomic profiling of animal models, researchers can systematically identify differentially expressed genes (DEGs) under various therapeutic interventions and elucidate their associated signaling pathways and biological functions (Jophlin et al., 2020).

In a carbon tetrachloride (CCl_4_)-induced liver fibrosis mouse model, Mercado-Gómez et al. revealed, via transcriptome analysis, the activation of multiple fibrosis-related pathways, particularly a marked upregulation of genes associated with protein polyubiquitination (Mercado-Gomez et al., 2020). This suggests a pivotal role for ubiquitin-mediated post-translational modification in the development of hepatic fibrosis. Key genes such as Col1a1, Col1a2, CD63, and Gpnmb were significantly upregulated, participating in extracellular matrix deposition, TGF-β signaling, and macrophage activation. Additionally, elevated ubiquitination of proliferating cell nuclear antigen (PCNA) implicated the involvement of DNA damage response (DDR) in hepatocyte injury repair and fibrogenesis. Transcriptomic data indicated that CCl_4_ exposure induced hepatocyte apoptosis and regeneration, accompanied by lipid metabolic disturbance and immune system activation, highlighting the systemic metabolic and inflammatory remodeling underlying liver fibrosis.

Zhu et al. employed RNA-seq and single-cell RNA-seq to further explore transcriptional regulation in a CCl_4_-induced liver fibrosis model (Zhu et al., 2024). Their findings showed that kaempferol significantly downregulated fibrogenic marker genes such as α-SMA, COL1A1, and TGF-β, and inhibited the Th17/IL-17 signaling pathway, which plays a key role in hepatic stellate cell (HSC) activation and proinflammatory cytokine production. Single-cell analysis revealed that kaempferol increased the proportions of CD4^+^ and CD8^+^ T cells, suggesting it may alleviate fibrosis progression by modulating the hepatic immune microenvironment.

In an AAV-HBV-infected mouse model, Kan et al. compared mRNA expression profiles in liver tissue at 1, 3, and 6 months post-infection via RNA-seq and identified 6,638 DEGs, among which 213 genes remained persistently upregulated across all time points (Kan et al., 2017). KEGG pathway analysis showed significant enrichment in pathways such as drug metabolism—cytochrome P450, chemical carcinogenesis, and carbon metabolism. Integrated transcriptomic and proteomic analysis identified 28 overlapping proteins, with four—Nlrp1b, Ankrd17, Traf3, and Nfkb1—participating in the RIG-I-like receptor signaling pathway, underscoring the role of oxidative stress and innate immunity in the fibrogenic process.

The development of hepatic fibrosis involves multiple mechanisms, including upregulation of extracellular matrix (ECM) genes, immune dysregulation, and metabolic reprogramming. Ubiquitination modulates protein stability and participates in injury repair and fibrotic deposition. The Th17/IL-17 axis and T cell dynamics shape the immune microenvironment. Meanwhile, metabolic rewiring and oxidative stress—mediated via cytochrome P450 and RIG-I signaling—bridge metabolism and immune responses. Multi-omics studies reveal the coordinated interactions among these mechanisms, offering a theoretical basis for targeted interventions.

### 3.2 Proteomics discoveries in animal models

Proteomics, as a central platform linking gene expression to cellular function, is an indispensable tool in exploring the mechanisms of hepatic fibrosis. Compared to transcriptomics, proteomics provides a more direct reflection of cellular functional responses, particularly in identifying alterations in metabolism, signaling activation, and post-translational modifications. High-throughput proteomic techniques in animal models allow researchers to comprehensively identify differentially expressed proteins and uncover potential antifibrotic targets and mechanisms (Cowan et al., 2010).

In a CCl_4_-induced liver fibrosis mouse model, Mercado-Gómez et al. used biotinylated ubiquitin-expressing transgenic mice (bioUb mice) and combined affinity purification with mass spectrometry (MS) to construct a hepatic ubiquitinome profile under fibrotic conditions (Mercado-Gomez et al., 2020). Their analysis revealed significant alterations in the ubiquitination of various substrates, notably PCNA, associated with DNA damage response. These ubiquitinated proteins also participated in apoptosis, lipid metabolism, cell survival, and regeneration, indicating that ubiquitination plays a bridging role across multiple biological processes in hepatic fibrosis.

In an AAV-HBV mouse model, Kan et al. used 2D-MALDI-TOF/TOF proteomics and identified 173 differentially expressed proteins, including CAT, PRDX1, GSTP1, NXN, and BLVRB, which were significantly elevated in the HBV-positive group and are closely associated with oxidative stress (Kan et al., 2017). KEGG pathway analysis revealed enrichment in prostate cancer, NOD-like receptor signaling, RIG-I-like receptor signaling, and apoptosis pathways. Protein-protein interaction (PPI) analysis showed that signaling pathways such as PI3K-Akt, AMPK, and Jak-STAT—related to cell proliferation, differentiation, apoptosis, and immune modulation—were activated during fibrosis.

In a CCl_4_-induced rat fibrosis model, Song et al. applied iTRAQ-based proteomics and identified 2,627 liver proteins, with 597 significantly differentially expressed between the gypenoside-treated and model groups (fold change ≥1.2 or ≤0.83) (Song et al., 2017). GO enrichment analysis indicated that these proteins were involved in redox regulation, amino acid metabolism, and glycolysis/gluconeogenesis, among others. Notably, three aldehyde dehydrogenases—ALDH1B1, ALDH2, and ALDH7A1—were upregulated in the gypenoside group, suggesting that the treatment may mitigate hepatic injury by enhancing acetaldehyde detoxification and reducing lipid peroxidation. Upregulation of PON1 implicated roles in cholesterol and steroid metabolism, while downregulation of S100A8 suggested anti-inflammatory effects. These results highlight the multi-targeted regulatory capacity of gypenoside in oxidative stress and inflammation.

Chen et al. employed data-independent acquisition (DIA) proteomics to investigate the molecular mechanisms of Zi Qi formula (ZQ) in a CCl_4_-induced mouse fibrosis model (Chen et al., 2024). Their results demonstrated significant upregulation of key glycolytic enzymes—HK2, PFKP, and PKM2—in fibrotic mice, which were suppressed by ZQ treatment. KEGG analysis indicated enrichment in glycolysis/gluconeogenesis and carbon metabolism pathways. Follow-up studies in liver sinusoidal endothelial cell (LSEC) models confirmed that ZQ inhibited glycolysis and reduced CXCL1 secretion, thereby limiting neutrophil infiltration and ameliorating liver inflammation and fibrotic deposition. These findings suggest that modulation of glycolytic activity in LSECs is a crucial antifibrotic mechanism of ZQ.

Proteomics reveals multilayered mechanisms of metabolic and immune regulation in hepatic fibrosis. Ubiquitination plays key roles in damage repair and ECM accumulation. Alterations in redox-associated enzymes reflect core aspects of metabolic reprogramming. Signaling pathways such as PI3K-Akt, AMPK, and Jak-STAT mediate cell growth and inflammation, while changes in glycolytic enzymes underscore the therapeutic potential of targeting metabolic pathways. Proteomics thus offers systematic evidence for understanding pathogenesis and identifying therapeutic targets.

### 3.3 Metabolomics discoveries in animal models

With the advancement of omics technologies, metabolomics emerges as a powerful tool for elucidating dynamic metabolic changes *in vivo* and is widely applied in the systematic investigation of liver fibrosis. By comprehensively mapping the metabolic reprogramming and its intricate relationship with immune and inflammatory responses, metabolomics offers critical insights into the systemic metabolic disturbances underlying liver fibrosis. Furthermore, these studies provide a solid theoretical foundation for the development of antifibrotic strategies targeting metabolism and immune modulation (Beyoğlu et al., 2024).

In a CCl_4_-induced mouse model of liver fibrosis, Mercado-Gómez et al. employed untargeted metabolomics to reveal marked metabolic reprogramming during fibrosis progression (Mercado-Gomez et al., 2020). Principal component analysis (PCA) demonstrated a clear separation between fibrotic and control groups, while volcano plots and heatmaps indicated a significant increase in phospholipids such as phosphatidylethanolamine, phosphatidylcholine, and lysophospholipids (LPC, LPE) in the fibrotic liver. These metabolites are closely associated with membrane synthesis, cell proliferation, and regeneration. Additionally, elevated levels of ceramides and sphingolipids suggested a potential role in hepatic injury repair or cell death pathways, supporting lipid remodeling as a hallmark of CCl_4_-induced fibrosis.

In another CCl_4_-induced fibrosis model, Zhu et al. reported that specific treatment significantly altered lipid metabolites such as 5-methylcytidine, LPI(18:0), and LPI(20:4), enriched in arachidonic acid metabolism and bile secretion pathways (Zhu et al., 2024). Fecal microbiota transplantation experiments confirmed that the antifibrotic effect was dependent on gut microbiota modulation, particularly the enrichment of probiotic genera such as Robinsoniella and Erysipelotrichaceae_UCG-003. These bacteria appeared to synergize with metabolic and transcriptional regulators in the liver, orchestrating lipid metabolism and inflammatory responses to delay fibrosis progression.

In a CCl_4_-induced rat model, Song et al. applied GC-MS-based metabolomics and detected 350 metabolic peaks, of which 17 were significantly altered in the treatment group (Song et al., 2017). Upregulated metabolites included acetate and ribonic acid, while glucose, asparagine, urea, and purines were downregulated. Integrated analysis with proteomics revealed enrichment in pathways such as glycolysis/gluconeogenesis, fructose and mannose metabolism, and glycine, serine, and threonine metabolism. The combined data suggested that energy metabolism disruption and glutathione-associated antioxidant imbalance are critical mechanisms in CCl_4_-induced fibrosis, which may be ameliorated by therapeutic intervention that restores metabolic homeostasis.

In another CCl_4_-induced mouse model, Chen et al. performed LC-MS on serum samples and identified multiple differential metabolites (Chen et al., 2024). Lactate, citrate, and glucose-6-phosphate (G6P) levels were significantly elevated in fibrotic mice but decreased following treatment. KEGG enrichment showed involvement of pathways such as phosphonate and phosphinate metabolism, nitrogen metabolism, and D-glutamine and D-glutamate metabolism. The intervention suppressed accumulation of glycolytic intermediates and attenuated metabolism-associated inflammatory responses, with proteomic data corroborating the metabolomics findings, highlighting a metabolite–immune axis underlying the antifibrotic effects.

In a separate CCl_4_-induced mouse model, Kong et al. used untargeted metabolomics to profile metabolic changes under therapeutic intervention (Kong et al., 2024). A total of 385 differential metabolites were identified, with bile acid dysregulation prominently observed in the fibrotic group, particularly elevated taurocholic acid, which decreased upon treatment. This metabolite showed positive correlation with proinflammatory gut microbiota such as Prevotella spp., suggesting a gut–liver axis involvement in metabolic inflammation. Enrichment analysis revealed that metabolites related to bile acid synthesis, biogenic amine metabolism, and branched-chain amino acid metabolism were significantly restored, supporting the notion that modulation of bile acid and amino acid networks contributes to fibrosis attenuation.

In a bile duct ligation (BDL)-induced model, Xi et al. performed fecal metabolomics and identified 18 fibrosis-related signature metabolites (Xi et al., 2024). Bile acids, including chenodeoxycholic acid (CDCA), taurochenodeoxycholic acid (TCDCA), deoxycholic acid (DCA), 3-dehydrocholic acid (3-DHC), and urobilinogen (UCA), were significantly elevated in BDL mice but reversed following intervention. Amino acid derivatives such as Thr-Ile-Arg and AHDOHA were significantly enriched in the treated group, suggesting potential roles in restoring gut–liver metabolic balance and cellular protection. Pathway analysis further highlighted bile acid metabolism, branched-chain amino acid metabolism, and lipid-redox pathways as key targets.

Overall, metabolomics studies reveal that lipid remodeling, aberrant glycolysis, and dysregulated amino acid metabolism are hallmarks of liver fibrosis, often accompanied by oxidative stress and compromised antioxidant defenses. Alterations in lipid metabolites not only reflect cellular repair demands but also modulate immune–inflammatory responses, emphasizing the role of the gut–liver axis in disease progression. Collectively, metabolomics provides a powerful framework for understanding metabolic imbalances in fibrosis and offers novel targets for therapeutic intervention.

### 3.4 Integrative multi-omics discoveries in animal models

In animal models, integrated multi-omics analysis provide unprecedented depth and scope for elucidating the pathological mechanisms of liver fibrosis (Dai and Shen, 2022). By combining transcriptomic, proteomic, and metabolomic data, researchers not only clarify the roles of different molecular layers in fibrogenesis but also uncovered how key signaling pathways interconnect and coordinate inflammation, metabolic dysfunction, and cellular remodeling (Baião et al., 2025).

At the transcriptional level, genes associated with extracellular matrix deposition, immune activation, and DNA damage repair are markedly upregulated during liver fibrosis. Pathways such as protein ubiquitination, Th17/IL-17 inflammatory signaling, TGF-β, and RIG-I-like receptor signaling are consistently activated (Kan et al., 2017; Mercado-Gomez et al., 2020; Zhu et al., 2024). Proteomic data further validate the functional consequences of these transcriptional changes, particularly through alterations in ubiquitinated proteins, upregulation of glycolytic enzymes, and enhanced activity of oxidative stress-related enzymes (Kan et al., 2017; Song et al., 2017; Mercado-Gomez et al., 2020; Chen et al., 2024). At the metabolomic level, studies reveal lipid remodeling, accumulation of glycolytic intermediates, and dysregulation of bile acid metabolism, all of which correlate closely with proteomic and transcriptomic findings—indicating dynamic crosstalk between these omic layers (Song et al., 2017; Mercado-Gomez et al., 2020; Chen et al., 2024; Kong et al., 2024; Xi et al., 2024; Zhu et al., 2024).

Pathway enrichment analyses highlight several central signaling cascades, including the TGF-β/Smad, PI3K-Akt, Jak-STAT, AMPK, RIG-I-like receptor, and Th17/IL-17 pathways. These pathways play pivotal roles in hepatic stellate cell (HSC) activation, inflammatory cell infiltration, and metabolic reprogramming. The persistent upregulation of glycolysis and lipid biosynthesis not only supports the energy and structural needs of fibrotic tissue but also exacerbates local inflammatory responses, forming a positive feedback loop. Moreover, some studies emphasize the importance of the gut-liver axis and microbiota-derived metabolites in regulating immune responses and hepatic metabolic homeostasis.

Multi-omics integration offers a comprehensive view of liver fibrosis as a complex, multi-pathway process driven by inflammation, metabolism, and immunity imbalance. The convergence of key signaling pathways across omics layers not only deepens our understanding of disease progression but also provides a solid theoretical foundation for developing combination therapies and identifying precise therapeutic targets (Maghsoudi et al., 2022; Zhao and Rhee, 2023).

## 4 Cross-species comparison between humans and animals

With the integration of transcriptomics, proteomics, and metabolomics, both human clinical cohorts and animal experimental models unveil a set of conserved biological processes driving liver fibrosis (Mercado-Gomez et al., 2020; Dong et al., 2023). Despite inherent species differences, a convergence in molecular signatures highlights key mechanisms underpinning hepatic fibrogenesis, providing cross-validational evidence for therapeutic target discovery. This cross-species alignment not only strengthens the pathophysiological relevance of animal models but also establishes a robust framework for translational research (Nevzorova et al., 2020). Building on this foundation, we integrated the multi-omics findings from **Table 1** (human studies) and **Supplementary Table 1** (animal studies), comparing commonalities and differences in key molecules, signaling pathways, and metabolic networks to explore their complementary value in elucidating disease mechanisms and facilitating clinical translation.

### 4.1 Transcriptomic comparison between humans and animals

Transcriptomic studies in both human patients and animal models reveal convergent molecular features, including hepatic stellate cell (HSC) activation, extracellular matrix (ECM) remodeling, and immune dysregulation. In human HBV-related liver fibrosis, genes such as COL1A1, TIMP1, ACTA2, and CTGF are consistently upregulated, reflecting structural reorganization and inflammatory signaling cascades (Dong et al., 2023). Similarly, animal models exposed to carbon tetrachloride (CCl_4_) show significant induction of ECM genes (e.g., Col1a1, Col1a2), accompanied by activation of pathways related to ubiquitination, Th17/IL-17 signaling, and cytochrome P450–mediated oxidative stress (Mercado-Gomez et al., 2020; Zhu et al., 2024). These overlapping findings suggest that transcriptomic reprogramming represents a conserved hallmark of fibrosis across species, despite differences in etiology and disease progression dynamics.

### 4.2 Proteomic comparison between humans and animals

Proteomic studies in both humans and animal models consistently reveal conserved mechanisms of liver fibrosis. In human HBV-related fibrosis, TIMP1, COL1A1, and ACTA2 are significantly upregulated and enriched in pathways associated with ECM architecture, cytoskeletal remodeling, and immune activation, corroborating transcriptomic findings (Dong et al., 2023). Correspondingly, in CCl_4_-induced fibrosis and AAV-HBV infection models, ECM proteins, cytoskeletal regulators, and oxidative stress–related enzymes (CAT, PRDX1, GSTP1) are likewise upregulated, while ubiquitination events such as PCNA modification link DNA damage response to fibrotic deposition (Kan et al., 2017). Interventional studies further suggest dynamic regulation of glycolytic enzymes and antioxidant pathways (Song et al., 2017; Chen et al., 2024). Overall, these proteomic signatures underscore the cross-species consistency of ECM remodeling, immune–metabolic reprogramming, and redox regulation, while the additional regulatory layers revealed in animal models enhance mechanistic clarity and translational relevance.

### 4.3 Metabolomic comparison between humans and animals

Metabolomic research highlights both convergences and divergences in human and animal liver fibrosis. In patients with chronic HCV infection, metabolic reprogramming involves amino acids (histidine, proline), choline, and lipid disturbances, which are closely associated with ECM production and bile acid dysregulation (Shanmuganathan et al., 2021). Lipidomic biomarker panels, particularly sphingomyelins and phosphatidylcholines, emerge as sensitive indicators to distinguish rapid from slow fibrosis progression (Cano et al., 2017). In contrast, animal models primarily recapitulate metabolic remodeling through lipid accumulation, aberrant glycolysis, and bile acid imbalance, often shaped by gut–liver axis interactions (Song et al., 2017; Mercado-Gomez et al., 2020; Chen et al., 2024; Kong et al., 2024; Zhu et al., 2024). In CCl_4_-induced models, elevated phosphatidylcholines, ceramides, and glycolytic intermediates mirror the lipid remodeling and energy stress observed in patients, while bile duct ligation (BDL) models reproduce bile acid overload resembling human cholestatic fibrosis (Xi et al., 2024). These metabolic disturbances in animal models—particularly lipid remodeling and bile acid dysregulation—are highly consistent with the metabolic signatures observed in human cohorts.

### 4.4 Cross-omics integration

Taken together, cross-species comparisons of transcriptomics, proteomics, and metabolomics highlight conserved molecular hallmarks of liver fibrosis, including HSC activation, ECM remodeling, immune–inflammatory signaling, oxidative stress, and metabolic dysregulation. Human data better capture the heterogeneity of etiology and genetic background, whereas animal models provide greater mechanistic clarity and validation of therapeutic interventions. The consistent transcriptomic, proteomic, and metabolomic alterations observed across human studies and animal models underscore conserved pathways that drive fibrosis progression. Such cross-species integration provides a powerful framework to validate conserved targets, reveal species-specific adaptations, and facilitate the translation of preclinical findings into clinically relevant biomarkers and therapeutic strategies.

## 5 Limitations

### 5.1 Human samples

Human liver fibrosis studies face substantial heterogeneity. Patients often present with chronic infections, metabolic comorbidities (such as type 2 diabetes, obesity, and metabolic syndrome), and diverse lifestyle factors (including alcohol consumption, diet, and physical activity), resulting in highly variable disease courses and significant differences in therapeutic responses and fibrosis progression. Genetic diversity—such as variations in PNPLA3 and TM6SF2—also influences metabolic status and susceptibility to fibrosis (Yigit et al., 2025).

Clinical studies typically rely on liver biopsy as the gold standard, but this is an invasive method that samples an extremely small portion of the liver (approximately 1/50,000 of the whole organ), leading to sampling errors and interobserver variability as high as 40–45% (Kawamura et al., 2022). Furthermore, ethical and practical constraints limit access to longitudinal samples. Variations in clinical staging, sampling locations, and pre-analytical processing can introduce batch effects, compromising the consistency and reproducibility of multi-omics analyses.

### 5.2 Animal models

Animal models predominantly rely on single injury induction methods (such as carbon tetrachloride [CCl_4_] injection, bile duct ligation, or chemical inducers), which result in rapid fibrosis progression and fail to fully capture the prolonged, multifactorial nature of human liver fibrosis (Wu et al., 2023). Current models cannot fully replicate the hepatic and systemic pathological features of human disease, as interspecies differences exist in immune cell composition, bile acid metabolism, and fibrogenic signaling pathways, thereby reducing the translational reliability of experimental results (Delire et al., 2015).

Moreover, a large body of preclinical data shows that the success rate of translating findings from animal models to human clinical applications remains below 10%, with failure rates exceeding 90% (Marshall et al., 2023). Laboratory animals are typically of uniform genetic background, which reduces individual variability for experimental control but fails to represent the genetic diversity of human populations, thus limiting the extrapolation of cross-species multi-omics analyses. Finally, animal studies employ highly standardized experimental designs, endpoint definitions, sampling time points, and tissue assessment methods, but these rarely align with human clinical cohorts (which often exhibit irregular sampling intervals and heterogeneous clinical stages). This mismatch hampers the cross-species comparability and consistency of multi-omics studies (Lee and Seki, 2023; Wu et al., 2023).

## 6 Future perspectives and translational significance

### 6.1 Future perspectives

The exponential growth of high-throughput omics technologies—including transcriptomics, proteomics, metabolomics, epigenomics, and microbiome profiling—provides unprecedented opportunities for decoding the complex mechanisms of liver fibrosis. Integrating these multi-omics datasets with advanced computational approaches, particularly machine learning (ML) and artificial intelligence (AI), is expected to substantially enhance biomarker discovery, disease stratification, and therapeutic response prediction. AI-driven methods, such as deep learning, network inference, and federated learning, can reconstruct intricate regulatory networks and simulate disease trajectories under different interventions, thereby enabling high-accuracy predictions (Beam and Kohane, 2018; Esteva et al., 2021). Recent studies demonstrate that ML models incorporating metabolomic, transcriptomic, and clinical parameters significantly outperform conventional clinical scoring systems in fibrosis staging (AUC up to 0.99) (Huang et al., 2017). Similarly, gut microbiome–based ML classifiers achieve robust diagnostic performance in distinguishing patients with fibrosis or cirrhosis from healthy controls (AUC ≈ 0.86) (Liu et al., 2023).

Future research should place greater emphasis on integrating emerging high-resolution technologies such as single-cell omics and spatial transcriptomics. These approaches enable the dissection of cellular heterogeneity, lineage trajectories, and microenvironmental interactions in liver fibrosis with unprecedented temporal and spatial resolution. For example, single-cell RNA sequencing (scRNA-seq) is widely applied to characterize the heterogeneity of hepatic stellate cells (HSCs), macrophages, and endothelial cells during liver fibrosis, as well as their dynamic changes throughout disease progression (Su et al., 2021; Yang et al., 2021; Cheng et al., 2023; Yang et al., 2023). Meanwhile, spatial transcriptomics techniques, such as MERFISH, further reveal the spatial distribution and gene expression patterns of cells in both healthy and fibrotic liver, providing new insights into the cellular and molecular mechanisms of liver fibrosis (Chung et al., 2022). The integration of single-cell and spatial technologies further uncovers regionalized molecular features in liver fibrosis, highlighting their translational potential for identifying spatial biomarkers and predicting therapeutic responses. Such approaches can capture dynamic changes in key cell populations, signaling networks, and fibrotic niches throughout disease initiation, progression, and regression. In parallel, establishing standardized, interoperable cross-species multi-omics databases is crucial for aligning human patient cohorts with well-annotated animal models, thereby enabling systematic comparisons of conserved and species-specific mechanisms (Cross et al., 2024; Wen and Ju, 2025).

Emerging experimental alternatives are garnering increasing attention, including three-dimensional (3D) culture systems such as organoids and liver-on-a-chip models. These models more accurately replicate the fibrotic process *in vitro*, encompassing extracellular matrix deposition, stellate cell activation, and bile acid dysregulation. Recent studies indicate that human liver organoids can simulate stellate cell activation and drug responses during fibrosis progression (Shao et al., 2025). Additionally, liver-on-a-chip technology, integrating microfluidic devices, reconstructs lobular architecture and hemodynamics, facilitating the evaluation of non-alcoholic fatty liver disease (NAFLD) and fibrosis therapeutics (Yang et al., 2023a). Notably, when these platforms are combined with AI-driven image analysis and predictive modeling, they not only serve as versatile platforms for drug screening and efficacy assessment but also reduce reliance on animal models, thereby enhancing clinical translation.

The future of liver fibrosis research lies in the synergistic integration of multi-omics technologies, advanced computational modeling, and physiologically relevant experimental platforms. High-throughput omics—including transcriptomics, proteomics, metabolomics, epigenomics, and microbiome profiling—combined with artificial intelligence (AI) and machine learning, enables more accurate biomarker discovery, disease stratification, and therapeutic prediction (Suo et al., 2025). Emerging single-cell and spatial omics approaches provide unprecedented resolution to dissect cellular heterogeneity, lineage dynamics, and microenvironmental interactions during fibrosis progression and regression (Li et al., 2025; Watson et al., 2025). Complementing these molecular insights, three-dimensional (3D) culture systems, including human liver organoids and liver-on-a-chip models, offer *in vitro* platforms that faithfully recapitulate fibrotic processes and support AI-driven drug screening and efficacy assessment (Mehta et al., 2024; Shao et al., 2025). Together, these advances promise to accelerate mechanistic understanding, facilitate translational studies, and ultimately improve patient-specific interventions in liver fibrosis.

### 6.2 Translational significance

From a translational perspective, multi-omics research offers a more systematic and comprehensive framework for preclinical drug screening and evaluation in liver fibrosis. By integrating transcriptomic, proteomic, and metabolomic data, researchers can elucidate the molecular, metabolic, and immune mechanisms of candidate antifibrotic drugs, revealing their effects on hepatocytes, hepatic stellate cells, and the immune microenvironment. This multilayered evaluation not only helps identify promising therapeutic compounds but also detects potential adverse or ineffective mechanisms early, thereby optimizing drug development pipelines and accelerating translation from bench to bedside (Schuppan et al., 2018).

Cross-species multi-omics comparisons further allow mechanistic findings from animal models to inform human studies, enabling the validation of molecular targets and biomarkers that are conserved across species. This approach improves the precision of clinical trial design and enhances the likelihood of success. In clinical applications, patient-specific multi-omics profiles provide a scientific basis for personalized treatment strategies, guiding drug or therapeutic combination selection according to individual patterns of gene expression, protein interaction networks, and metabolic signatures. Such strategies can improve efficacy, reduce unnecessary treatments, and ultimately contribute to better long-term outcomes for patients with liver fibrosis and cirrhosis (Nevzorova et al., 2020).

## 7 Conclusion

In summary, this review integrates multi-omics data to elucidate the key mechanisms of liver fibrosis and cirrhosis, characterized by hepatic stellate cell activation, extracellular matrix deposition, immune–inflammatory dysregulation, and metabolic reprogramming. By analyzing transcriptomic, proteomic, and metabolomic data from both human and animal models, we identified conserved molecular signatures and key pathways driving fibrosis progression. These insights not only enhance our understanding of the disease but also support key translational outcomes. Specifically, multi-omics approaches enable the development of improved diagnostic panels, identification of stratification biomarkers, and prioritization of therapeutic targets, paving the way for precision medicine and personalized medicine.
