# Supplementary material for: Liver fibrosis and cirrhosis in the multi-omics era: mechanisms and therapeutic perspectives from human and animal models
Source: Front Cell Infect Microbiol. 2026 Jan 8;15:1686649. doi: 10.3389/fcimb.2025.1686649 (PMC12823897; doi:10.3389/fcimb.2025.1686649)
Supplement: Supplementary file 1 [file Table1.docx]

**Supplementary Table 1: Multi-omics studies on animal models of hepatic fibrosis and cirrhosis**

| Author, country, publication year (ref) | Species/gender/age or weight | Modeling methods (annotation method/dose/frequency/duration) | Intervention (annotation method/dose/frequency/duration) | Hepatic biochemical profile | Assays/platform | Omics | Case vs. Control | Comparison | Statistics | Major findings | Key pathway | Validation | Database number |
| --- | --- | --- | --- | --- | --- | --- | --- | --- | --- | --- | --- | --- | --- |
| Chen, China, 2024[36] | C57BL/6 mice/male/6 wks/ 18-22g | ZQ-Low group: Intraperitoneal Injection/ 0.5mL/kg CCl4(10% olive oil)/ three times per week/ 12wks | Intragastric Gavage 8.8 g/kg ZQ/ daily/ weeks 9-12 | ALT:125±50 | BCA and LC-MS/MS / NanoElute and Metascape | Proteomics | 6 vs.6 | ZQ CCl4 mice vs.CCl4 mice | Student's t-test/One-Way ANOVA/Tukey's test/FC | 1.The expression levels of HK2, PFKP, and PKM2 initially increased in the model group, but decreased after ZQ treatment. | 1.Metabolic pathway | 1. WB | UniProt-Mus musculus-10090-2019-10 |
|  |  |  |  | AST:320±50 |  |  |  |  |  |  | 2.Carbon metabolism | 2.RT-qPCR |  |
|  |  |  |  | ALP:140±10 |  |  |  |  |  |  | 3.Glycolysis/ Gluconeogenesis |  |  |
|  |  |  |  | HA:70±10 |  |  |  |  |  |  |  |  |  |
|  |  |  |  | LN:600±100 |  |  |  |  |  |  |  |  |  |
|  |  |  |  | PC-III:7±2 |  |  |  |  |  |  |  |  |  |
|  |  | ZQ-Middle group: Intraperitoneal Injection/ 0.5mL/kg CCl4(10% olive oil) / three times per week/ 12wks | Intragastric Gavage/ 17.6 g/kg ZQ/ daily/ weeks 9-12 | ALT:80±100 |  |  |  |  |  | 2.ZQ may play a role in inhibiting glycolytic metabolism in mice with liver fibrosis. |  |  |  |
|  |  |  |  | AST:280±50 |  |  |  |  |  |  |  |  |  |
|  |  |  |  | ALP:125±10 |  |  |  |  |  |  |  |  |  |
|  |  |  |  | HA:45±10 |  |  |  |  |  |  |  |  |  |
|  |  |  |  | LN:400±100 |  |  |  |  |  |  |  |  |  |
|  |  |  |  | PC-III:6±2 |  |  |  |  |  |  |  |  |  |
|  |  | ZQ-High group: Intraperitoneal Injection/ 0.5mL/kg CCl_4_(10% olive oil)/ three times per week/ 12wks | Intragastric Gavage 35.2 g/kg ZQ/ daily/ weeks 9-12 | ALT:80±100 | LC-MS / UPLC and Q-Exactive | Metabolomics | 6 vs.6 vs.6 | ZQ CCl_4_ mice vs.CCl_4_ mice vs.Ctrl mice | Student's t-test/One-Way ANOVA/PCA/OPLS-DA | 1.The ZQ treatment significantly reduced the levels of MPO in liver tissues, indicating that ZQ decreased neutrophil infiltration by inhibiting the secretion of CXCL1. | 1.Phosphonate and phosphinate metabolism | IF | None |
|  |  |  |  | AST:270±25 |  |  |  |  |  |  | 2.Nitrogen metabolism |  |  |
|  |  |  |  | ALP:130±25 |  |  |  |  |  |  | 3.D-Glutamine and D-glutamate metabolism |  |  |
|  |  |  |  | HA:50±10 |  |  |  |  |  |  |  |  |  |
|  |  |  |  | LN:420±100 |  |  |  |  |  |  |  |  |  |
|  |  |  |  | PC-III:5±2 |  |  |  |  |  |  |  |  |  |
|  |  | Model group: Intraperitoneal Injection/ 0.5mL/kg CCl_4_(10% olive oil)/ three times per week/ 12wks | None | ALT:160±100 |  |  |  |  |  | 2.ZQ could potentially modulate LSEC glycolysis and subsequently inhibit neutrophil infiltration in liver fibrosis. |  |  |  |
|  |  |  |  | AST:440±100 |  |  |  |  |  |  |  |  |  |
|  |  |  |  | ALP:170±10 |  |  |  |  |  |  |  |  |  |
|  |  |  |  | HA:115±10 |  |  |  |  |  |  |  |  |  |
|  |  |  |  | LN:750±50 |  |  |  |  |  |  |  |  |  |
|  |  |  |  | PC-III:9±1 |  |  |  |  |  |  |  |  |  |
|  |  | Control group: Intraperitoneal Injection/ saline(equal volume)/ three times per week/ 12wks | None | ALT:25±10 |  |  |  |  |  |  |  |  |  |
|  |  |  |  | AST:100±10 |  |  |  |  |  |  |  |  |  |
|  |  |  |  | ALP:75±10 |  |  |  |  |  |  |  |  |  |
|  |  |  |  | HA:25±10 |  |  |  |  |  |  |  |  |  |
|  |  |  |  | LN:220±25 |  |  |  |  |  |  |  |  |  |
|  |  |  |  | PC-III:4±1 |  |  |  |  |  |  |  |  |  |
|  |  |  |  |  |  |  |  |  |  |  |  |  |  |
| Kong, China, 2024[38] | C57BL/6 mice/male/8wks | Metformin group: Intraperitoneal injection/ CCl₄ (1:3 olive oil, 4 μl/g)/ twice times per week/ 6wks | Intragastric gavage/ 0.1% metformin/ twice times per week/ 6wks | AST:250±25 | LC-MS / UPLC and Q-Exactive | Metabolomics | 10 vs.10 | Metformin mice vs.Model mice | Student's t-test/One-Way ANOVA/Dunnett’s T3/FC | 1.Metformin may play an anti-fibrosis role by inhibiting Prevotella and reducing taurocholic acid secretion. | 1.Linoleic acid metabolism | qRT-PCR | None |
|  |  |  |  | ALT:150±25 |  |  |  |  |  |  | 2.Amoebiasis |  |  |
|  |  |  |  | PC-III:8±1 |  |  |  |  |  |  | 3.Purine metabolism |  |  |
|  |  |  |  | IV-C:35±2 |  |  |  |  |  |  |  |  |  |
|  |  |  |  | HA:0.20±0.02 |  |  |  |  |  |  |  |  |  |
|  |  |  |  | LN:700 ± 50 |  |  |  |  |  |  |  |  |  |
|  |  | Model group: Intraperitoneal injection/ CCl₄ (1:3 olive oil, 4 μl/g)/ twice times per week/ 6wks | None | AST:400±25 |  |  |  |  |  | 2.Metformin exerts anti-fibrotic effects by regulating the TGF-β/Smads and TIMP-1/MMPs signaling pathways, thereby inhibiting hepatocyte apoptosis. |  |  |  |
|  |  |  |  | ALT:250±30 |  |  |  |  |  |  |  |  |  |
|  |  |  |  | PC-III:9±1 |  |  |  |  |  |  |  |  |  |
|  |  |  |  | IV-C:38±2 |  |  |  |  |  |  |  |  |  |
|  |  |  |  | HA:0.23±0.01 |  |  |  |  |  |  |  |  |  |
|  |  |  |  | LN:800±50 |  |  |  |  |  |  |  |  |  |
|  |  | Control group: Intraperitoneal injection/ saline(equal volume)/ twice times per week/ 6wks | None | AST:100±5 |  |  |  |  |  |  |  |  |  |
|  |  |  |  | ALT:20±5 |  |  |  |  |  |  |  |  |  |
|  |  |  |  | PC-III:7±1 |  |  |  |  |  |  |  |  |  |
|  |  |  |  | IV-C:30±1 |  |  |  |  |  |  |  |  |  |
|  |  |  |  | HA:0.15±0.03 |  |  |  |  |  |  |  |  |  |
|  |  |  |  | LN:600±50 |  |  |  |  |  |  |  |  |  |
|  |  |  |  |  |  |  |  |  |  |  |  |  |  |
| Xi, China, 2024[39] | C57BL/6 mice/male/10wks | BDL+AG 25 mg/kg group: BDL | Oral Gavage/ 25 mg/kg AG/ daily at 9–10 am/ 2wks | ALT:400±50 | LC-MS/MS / NMR | Metabolomics | 6 vs.6 vs.6 | BDL+AG mice vs.BDL mice vs.Sham mice | One-Way ANOVA/PCA/OPLS-DA | 1.AG treatment could retard the fibrotic process in the liver, and reduce inflammation infiltration and bile duct damage. | 1.Microbial metabolism in diverse environments | WB | ERP109777 |
|  |  |  |  | AST:40±10 |  |  |  |  |  |  | 2.Biosynthesis of secondary metabolites |  |  |
|  |  |  |  | TBIL:500±50 |  |  |  |  |  |  | 3.Biosynthesis of antibiotics |  |  |
|  |  |  |  | ALP:500±100 |  |  |  |  |  |  |  |  |  |
|  |  |  |  | LPS:700±10 |  |  |  |  |  |  |  |  |  |
|  |  | BDL+AG 100mg/kg group: BDL | Oral Gavage/ 100 mg/kg AG/ daily at 9–10 am/ 2wks | ALT:100±10 |  |  |  |  |  | 2.During the process of AG tea improving liver fibrosis, bile acid derivatives (such as CDCA, TCDCA, 3-DHC, UCA, DCA, etc.) play significant roles. |  |  |  |
|  |  |  |  | AST:20±5 |  |  |  |  |  |  |  |  |  |
|  |  |  |  | TBIL:100±10 |  |  |  |  |  |  |  |  |  |
|  |  |  |  | ALP:100±10 |  |  |  |  |  |  |  |  |  |
|  |  |  |  | LPS:500±10 |  |  |  |  |  |  |  |  |  |
|  |  | BDL group: BDL | Oral Gavage/ saline(equal volume)/ daily at 9–10 am/ 2wks | ALT:500±100 |  |  |  |  |  | 3.After AG tea treatment, the relative abundance of beneficial bacteria increased, while the relative abundance of harmful bacteria decreased. |  |  |  |
|  |  |  |  | AST:50±10 |  |  |  |  |  |  |  |  |  |
|  |  |  |  | TBIL:600±100 |  |  |  |  |  |  |  |  |  |
|  |  |  |  | ALP:600±100 |  |  |  |  |  |  |  |  |  |
|  |  |  |  | LPS:800±10 |  |  |  |  |  |  |  |  |  |
|  |  | Sham group: sham surgery | Oral Gavage/ saline(equal volume)/ daily at 9–10 am/ 2wks | ALT:10±1 |  |  |  |  |  |  |  |  |  |
|  |  |  |  | AST:10±1 |  |  |  |  |  |  |  |  |  |
|  |  |  |  | TBIL:10±1 |  |  |  |  |  |  |  |  |  |
|  |  |  |  | ALP:10±1 |  |  |  |  |  |  |  |  |  |
|  |  |  |  | LPS:450±10 |  |  |  |  |  |  |  |  |  |
|  |  |  |  |  |  |  |  |  |  |  |  |  |  |
| Zhu, China, 2024[33] | Balb/c mice/male/4wks | Kaempferol (10 mg/ kg) group: Intraperitoneal injection/ CCl4(1:1 in corn oil, 1 ml/kg)/ daily/ 4wks | Oral Gavage/ Kaempferol (10 mg/kg) with saline and CCl₄ (1:1 volume)/ daily/ 4wks | ALT:250±20 | Illumina NovaSeq 6000 system/Chromium | Transcriptomics | 10 vs.10 | Kaempferol mice vs. CCl4 mice | ANOVA | 1.Kaempferol significantly increased the proportions of CD4+ T cells and CD8+ T cells, activated immune cells, and enhanced immune function, thereby protecting the body and controlling inflammation. | 1.Systemic lupus erythematosus | 1,RNA-Seq | None |
|  |  |  |  | AST:250±20 |  |  |  |  |  |  | 2.Complement and coagulation cascades | 2.qPCR |  |
|  |  |  |  | TBIL:14±1 |  |  |  |  |  |  | 3.Influenza A | 3.IHC |  |
|  |  |  |  | ALB:18±1 |  |  |  |  |  |  | 4.Ovarian steroidogenesis |  |  |
|  |  |  |  | PT:125±20 |  |  |  |  |  |  |  |  |  |
|  |  |  |  | HA:210±10 |  |  |  |  |  |  |  |  |  |
|  |  |  |  | PC-III:30±2 |  |  |  |  |  |  |  |  |  |
|  |  |  |  | LN:200±10 |  |  |  |  |  |  |  |  |  |
|  |  | Kaempferol (20 mg/ kg) group: Intraperitoneal injection/ CCl4(1:1 in corn oil, 1 ml/kg)/ daily/ 4wks | Oral Gavage: Kaempferol (20 mg/kg) with saline and CCl₄ (1:1 volume)/ daily/ 4wks | ALT:200±10 |  |  |  |  |  | 2.Kaempferol exerted anti-inflammatory and immune-enhancing effects in PDGF-induced LX2 cells by downregulating the Th17/IL-17 signaling pathway. |  |  |  |
|  |  |  |  | AST:200±10 |  |  |  |  |  |  |  |  |  |
|  |  |  |  | TBIL:15±3 |  |  |  |  |  |  |  |  |  |
|  |  |  |  | ALB:20±1 |  |  |  |  |  |  |  |  |  |
|  |  |  |  | PT:150±5 |  |  |  |  |  |  |  |  |  |
|  |  |  |  | HA:190±10 |  |  |  |  |  |  |  |  |  |
|  |  |  |  | PC-III:25±2 |  |  |  |  |  |  |  |  |  |
|  |  |  |  | LN:160±20 |  |  |  |  |  |  |  |  |  |
|  |  | Kaempferol (40 mg/ kg) group: Intraperitoneal injection/ CCl4(1:1 in corn oil, 1 ml/kg)/ daily/ 4wks | Oral Gavage/ Kaempferol (40 mg/kg) with saline and CCl₄ (1:1 volume)/ daily/ 4wks | ALT:190±20 | (MS/MS) / Illumina NovaSeq 6000 system | Metabolomics | 10 vs.10 | Kaempferol mice vs. CCl4 mice | Student's t-test/PCA/OPLS-DA | 1.In the CCl₄-induced liver fibrosis mouse model, levels of glycerophospholipid and sphingolipid metabolites are significantly elevated. | 1.Glycine, serine and threonine metabolism | FMT | None |
|  |  |  |  | AST:190±20 |  |  |  |  |  |  | 2.Arginine and prolinemetabolism |  |  |
|  |  |  |  | TBIL:17±2 |  |  |  |  |  |  | 3.Choline metabolism in cancer |  |  |
|  |  |  |  | ALB:22±1 |  |  |  |  |  |  | 4.Pentose phosphate pathway |  |  |
|  |  |  |  | PT:160±10 |  |  |  |  |  |  |  |  |  |
|  |  |  |  | HA:150±10 |  |  |  |  |  |  |  |  |  |
|  |  |  |  | PC-III:20±2 |  |  |  |  |  |  |  |  |  |
|  |  |  |  | LN:150±20 |  |  |  |  |  |  |  |  |  |
|  |  | Colchicine-treated group: Intraperitoneal injection/ CCl4(1:1 in corn oil, 1 ml/kg)/ daily/ 4wks | Oral Gavage/ 0.2 mg/kg colchicine/ daily/4wks | ALT:220±10 |  |  |  |  |  | 2.Glycyrrhizol mitigates CCl₄-induced liver fibrosis by modulating gut microbiota and metabolite levels, particularly by enriching beneficial bacteria and regulating lipid metabolism. |  |  |  |
|  |  |  |  | AST:210±20 |  |  |  |  |  |  |  |  |  |
|  |  |  |  | TBIL:15±2 |  |  |  |  |  |  |  |  |  |
|  |  |  |  | ALB:20±1 |  |  |  |  |  |  |  |  |  |
|  |  |  |  | PT:125±25 |  |  |  |  |  |  |  |  |  |
|  |  |  |  | HA:200±10 |  |  |  |  |  |  |  |  |  |
|  |  |  |  | PC-III:30±2 |  |  |  |  |  |  |  |  |  |
|  |  |  |  | LN:200±10 |  |  |  |  |  |  |  |  |  |
|  |  | CCl_4_ group: Intraperitoneal Injection/ CCl4(1:1 in corn oil, 1 ml/kg)/ daily/ 4wks | Oral Gavage/ saline(equal volume)/ daily/ 4wks | ALT:300±10 |  |  |  |  |  |  |  |  |  |
|  |  |  |  | AST:300±10 |  |  |  |  |  |  |  |  |  |
|  |  |  |  | TBIL:25±4 |  |  |  |  |  |  |  |  |  |
|  |  |  |  | ALB:15±1 |  |  |  |  |  |  |  |  |  |
|  |  |  |  | PT:100±10 |  |  |  |  |  |  |  |  |  |
|  |  |  |  | HA:250±20 |  |  |  |  |  |  |  |  |  |
|  |  |  |  | PC-III:40±2 |  |  |  |  |  |  |  |  |  |
|  |  |  |  | LN:250±20 |  |  |  |  |  |  |  |  |  |
|  |  | Control group:  same corn oil/ daily/ 4wks | Oral Gavage/ saline(equal volume)/ daily/ 4wks | ALT:100±10 |  |  |  |  |  |  |  |  |  |
|  |  |  |  | AST:100±20 |  |  |  |  |  |  |  |  |  |
|  |  |  |  | TBIL:5±1 |  |  |  |  |  |  |  |  |  |
|  |  |  |  | ALB:30±2 |  |  |  |  |  |  |  |  |  |
|  |  |  |  | PT:175±10 |  |  |  |  |  |  |  |  |  |
|  |  |  |  | HA:100±10 |  |  |  |  |  |  |  |  |  |
|  |  |  |  | PC-III:15±1 |  |  |  |  |  |  |  |  |  |
|  |  |  |  | LN:100±10 |  |  |  |  |  |  |  |  |  |
|  |  |  |  |  |  |  |  |  |  |  |  |  |  |
| Mercado-Gómez, Spain, 2020[32] | Mice/female | Intraperitoneal Injection/ (0.2mL/kg)CCl4/ three times per week/ 8wks | None | NA | Gene Chip Analysis / Affymetrix GeneChip | Transcriptomics | 13 vs.9 | CCl4 mice vs. Ctrl mice | Student's t-test | 1.After 6 weeks of CCl₄-induced liver fibrosis, we found increased expression of genes encoding E3 ubiquitin-protein ligases Cbl and Arih1. | 1.Ubiquitination Pathway | qPCR | GSE141821 |
|  |  |  |  |  |  |  |  |  |  |  | 2.Extracellular Matrix Pathway |  |  |
|  | BioUb mice | Intraperitoneal Injection/ (0.6mL/kg)CCl4/ once per week/ 6wks | None | NA | BCA / LC-MS/MS | Proteomics | 5 vs.5 | BioUb CCl4 mice vs. BioUb Ctrl mice |  | 2.Ubiquitination is crucial for regulating cell death, survival, function, lipid metabolism, and DNA repair in CCl₄-induced liver fibrosis. | 1.Glycolysis / Gluconeogenesis | WB | None |
|  |  |  |  |  |  |  |  |  |  |  | 2.Fructose and mannose metabolism |  |  |
|  |  |  |  |  |  |  |  |  |  |  | 3.Glycine, serine and threonine metabolism |  |  |
|  | C57BL/6 mice/male/3mos | Intraperitoneal Injection/ (0.6mL/kg)CCl4/ once per week/ 6wks | None | NA | LC-MS / LC-TOF-MS | Metabolomics | 4 vs.5 | C57BL/6 CCl4 mice vs.C57BL/6 Ctrl mice |  | 3.CCl₄-induced liver fibrosis significantly alters hepatic metabolism, with notable increases in glycerophospholipids, ceramides, and sphingolipids. | 1.Carbohydrate metabolism | Gene Expression Validation | None |
|  |  |  |  |  |  |  |  |  |  |  | 2.Amino acid metabolism |  |  |
|  |  |  |  |  |  |  |  |  |  |  | 3.Lipid metabolism |  |  |
|  |  |  |  |  |  |  |  |  |  |  |  |  |  |
| Song, China, 2017[35] | Wistar rats/male/180-200g | Gypenoside group: Intraperitoneal injection/ 1:1(ccl4:2ml/kg olive oil)/ twice times per week/ 9wks | Intragastric Administration/ water or 200 mg/kg gypenoside/ daily/ weeks 6-9 | Hyp:400±100 | BCA,iTRAQ and MS / 4800 MALDI TOF/TOF | Proteomics | 10 vs.10 | CCl4+gypenoside Wistar vs.CCl4 Wistar | One-Way ANOVA | 1.The upregulation of ALDH proteins may alleviate liver fibrosis by enhancing aldehyde metabolism and reducing oxidative stress. | 1.Oxidation-reduction | WB | PXD005267 |
|  |  |  |  |  |  |  |  |  |  |  | 2.Amino acid metabolism |  |  |
|  |  |  |  |  |  |  |  |  |  |  | 3.Glycolysis/ gluconeogenesis |  |  |
|  |  | Model group: Intraperitoneal injection/ 1:1(ccl4:2ml/kg olive oil)/ twice times per week/ 9wks | None | Hyp:500±50 |  |  | 10 vs.10 vs.10 | CCl4+gypenoside Wistar vs.CCl4 Wistar vs.Ctrl Wistar |  | 2.Gypenoside significantly ameliorated CCl₄-induced liver fibrosis by modulating glycolysis metabolism and protecting cells from aldehyde and lipid peroxidation damage. |  |  |  |
|  |  | Control group: Intraperitoneal injection/ saline(equal volume)/ twice times per week/ 9wks | None | Hyp:100 ±10 | GC-MS / Agilent 6890 GC system coupled with 5975B mass spectrometer | Metabolomics | 10 vs.10 | CCl_4_+gypenoside Wistar vs.CCl_4_ Wistar | PCA/PLS-DA/OPLS | 3.Gypenoside improved liver histopathology in CCl₄-induced fibrosis rats, likely via altering glycolysis metabolism and protecting against aldehyde and LPO damage by upregulating ALDH. | 1.Glycolysis/ Gluconeogenesis | 1.H&E | MTBLS123 |
|  |  |  |  |  |  |  |  |  |  |  | 2.Fructose and Mannose Metabolism | 2.Sirius Red |  |
|  |  |  |  |  |  |  |  |  |  |  | 3.Glycine, Serine, and Threonine Metabolism |  |  |
|  |  |  |  |  |  |  |  |  |  |  |  |  |  |
| Kan, China, 2017[17] | C57BL/6 mice/male/6-8wks | None | 1mo HBV group: Tail Vein Injection/ 200 μl of rAAV8-HBV 1.2 vector(2 × 1011vg)/ once at the start/ 1mo | Hyp:120±10 | RNA-Seq / Illumina HiSeq | Transcriptomics | 4 vs.4 | HBV(-) vs.  HBV(+) | DESeq | 1.Redox reactions and innate immunity are important in liver fibrosis. | 1.Drug metabolism - cytochrome P450 | RT-qPCR | GSE95424 |
|  |  |  |  |  |  |  |  |  |  |  | 2.Chemical carcinogenesis |  |  |
|  |  |  |  |  |  |  |  |  |  |  | 3.Carbon metabolism |  |  |
|  |  | None | 3mos HBV group: Tail Vein Injection/ 200 μl of rAAV8-HBV 1.2 vector(2 × 1011 vg)/ once at the start/ 3mos | Hyp:160±10 |  |  |  |  |  | 2.Glutathione metabolism is closely related to intracellular reactive oxygen species metabolism, a major factor in liver fibrosis. |  |  |  |
|  |  | None | 6mos HBV group: Tail Vein Injection/ 200 μl of rAAV8-HBV 1.2 vector(2 × 1011 vg)/ once at the start/ 6mos | Hyp:220±10 | BCA,iTRAQ and 2-DE / MALDI-TOF/TOF and MS | Proteomics | 4 vs.4 | HBV(-) vs.  HBV(+) | Student's t-test/ANOVA | 3.HSCs are crucial in liver fibrosis, with their activation being a key event. Activated HSCs are the main source of extracellular matrix and cytokines, which regulate the fibrosis process. | 1.Prostate cancer | WB | None |
|  |  | None | Control group: Tail Vein Injection/ PBS(equal volume)/ once at the start/ 1,3 and 6mos | Hyp:120±10 |  |  |  |  |  |  | 2.NOD-like receptor signaling pathway |  |  |
|  |  |  |  |  |  |  |  |  |  |  | 3.RIG-I-like receptor signaling pathway |  |  |
|  |  |  |  |  |  |  |  |  |  |  | 4.Apoptosis |  |  |

^a^Hepatic biochemical profile is presented as "(mean ± SD)".

Units: Hyp,μg/g; ALT,U/L ; AST,U/L ; ALP,U/L ; HA,μg/L; LN,μg/L; PC-III,μg/L; IV-C,ng/mL; TBIL,μmol/L ; LPS,ng/L; ALB,g/L ; PT,ng/L.

Abbreviations: AG, Ampelopsis grossedentata; ALB, albumin; ALP, alkaline phosphatase; ALT, alanine aminotransferase; ANOVA, analysis of variance; AST, Aspartate Aminotransferase; BCA, Bicinchoninic Acid Assay; BDL, Bile Duct Ligation; FC, Fold Change; FMT, Fecal Microbiota Transplantation; HA, Hyaluronic Acid; H&E, Hematoxylin and Eosin; Hyp, hydroxyproline; IF, Immunofluorescence; IHC, Immunohistochemistry; IV-C, Type IV Collagen; LN, Laminin; LPS, Lipopolysaccharide; mos, months; NA, not available; NMR, maximum neighborhood component; OPLS-DA, Orthogonal Partial Least Squares Discriminant Analysis; PCA, principal component analysis; PC-III, Procollagen Type III; PLS-DA, Partial Least Squares Discriminant Analysis; PT, Prothrombin; qRT, quantitative real-time; TBIL, total bilirubin; TOF, time-of-flight; UPLC, Ultra-Performance Liquid Chromatography; WB, western blot; wks, weeks.
